# Characterization of the Venom of *C. d. cumanesis* of Colombia: Proteomic Analysis and Antivenomic Study

**DOI:** 10.3390/toxins10020085

**Published:** 2018-02-17

**Authors:** Juan Carlos Quintana-Castillo, Leidy Johana Vargas, Cesar Segura, Sebastián Estrada-Gómez, Julio César Bueno-Sánchez, Juan Carlos Alarcón

**Affiliations:** 1 School of Medicine, Universidad Cooperativa de Colombia, Sede Medellín, Street 50 A N° 41-20, Medellín 050010, Colombia; leidy.vargasmu@campusucc.edu.co; 2 Malaria Group, School of Medicine, University of Antioquia UdeA, Street 70 N° 52-21, Medellín 050010, Colombia; cesars79@gmail.com; 3 Ophidism/Scorpionism Program, Food and Pharmaceutical Sciences Faculty, University of Antioquia UdeA, Street 70 N° 52-21, Medellín 050010, Colombia; sebastian.estrada@udea.edu.co (S.E.-G.); juan.alarcon@udea.edu.co (J.C.A.); 4 Reproduction Group, School of Medicine, University of Antioquia UdeA, Street 70 N° 52-21, Medellín 050010, Colombia; julio.bueno@yahoo.com

**Keywords:** *C. d. cumanensis* venom, antivenom, HPLC-nESI-MS/MS, peptide identification, This work carried out a proteomic analysis of *C. d. cumanensis* from Colombia by using HPLC-nESI MS/MS and subsequently was evaluated the effect of a commercial antivenom against the venom

## Abstract

The Colombian rattlesnake *Crotalus durissus cumanensis* is distributed in three geographic zones of the country: the Atlantic Coast, the upper valley of the Magdalena River, and the eastern plains of the Colombian Orinoquía. Its venom induces neurological symptoms, such as eyelid ptosis, myasthenic facies, and paralysis of the respiratory muscles, which can lead to death. Identification and analysis of *C. d. cumanensis* showed nine groups of proteins responsible for the neurotoxic effect, of which the crotoxin complex was the most abundant (64.71%). Immunorecognition tests of *C. d. cumanensis* showed that the use of a commercial antivenom manufactured in Mexico resulted in immunoreactivity.

## 1. Introduction

The group under the generic name “rattlesnake” (*Crotalus*) constitutes a monophyletic group of snakes next to the genus *Sistrurus*. It is characterized by the presence of a cornified structure derived from the ecdysis (molting) in its tail and by its wide geographic distribution from the southeast of Canada to the north of Argentina. This genus includes the species *Crotalus durissus* (*C. d.*), with at least 11 subspecies closely related to each other and a wide distribution in the American neotropics [1,2].

**Figure 1:**
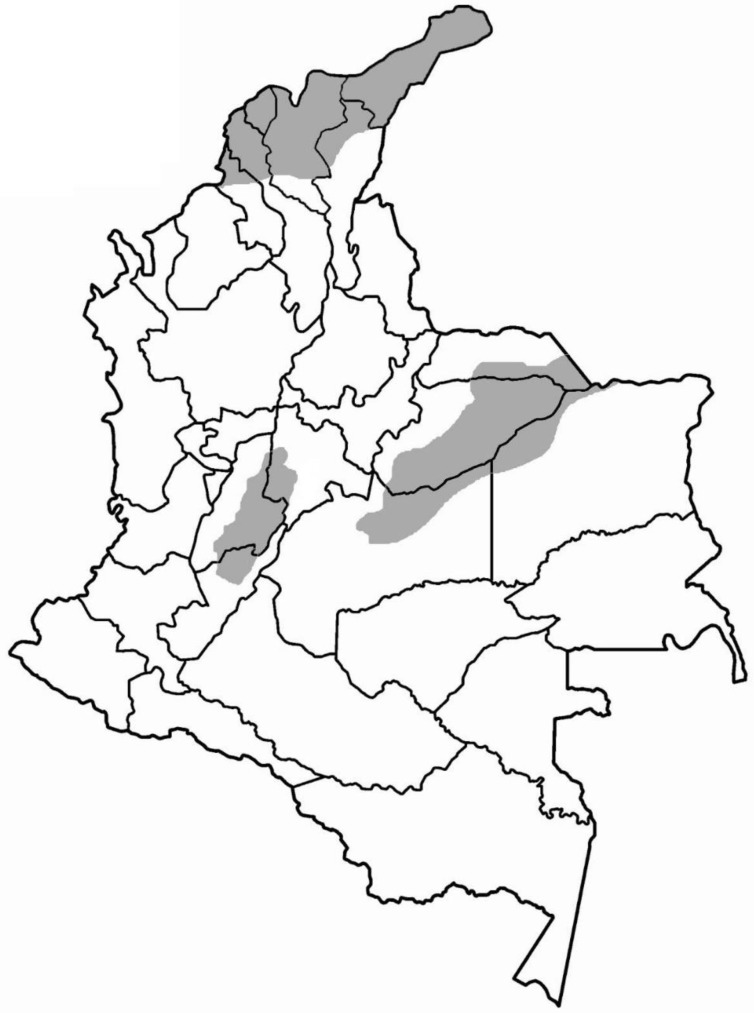
Distribution of *C. d. cumanensis* in Colombia.

*C. d. cumanensis* is found in Colombia, specifically in dry or semidry forest areas (up to 1000 m.a.s.l.) of the eastern plains (departments of Meta, Casanare, and Vichada), the north of the country (departments of Guajira, Bolívar, Magdalena, Atlántico, Córdoba, Sucre, and Cesar), and the upper and middle part of the Magdalena River valley (departments of Huila, Tolima, Cundinamarca, and Caldas); however, there are reports of areas above 2000 m.a.s.l. in the Sierra Nevada de Santa Marta [1] (see Figure 1).

The venom of the South American rattlesnake is composed of a complex mixture of peptides, enzymes, and toxins. Regarding toxins, crotamine, gyroxine, convulxin, a thrombin-like enzyme [3,4], and the crotoxin complex (composed of two subunits, A and B), which corresponds to a heterodimeric PLA_2_ and can make up between 70% and 80% of the toxin content of the venom [5,6], are responsible for the high neurotoxic, nephrotoxic, and myotoxic activity [4,7,8].

Envenomation by South American rattlesnakes induces neurotoxic symptoms due to the venom’s ability to interrupt neuromuscular connectivity, leading to a progressive paralysis that generates symptoms such as palpebral ptosis, myastenic facies, and progressive flaccid paralysis, and even respiratory failure that may result in the death of the victim. In addition, envenomation is accompanied, in some cases, by nephrotoxicity due to tubular necrosis, renal failure, and rhabdomyolysis, which further complicates the clinical picture [7,9,10,11,12,13,14]. The objective of this work was to identify and characterize the protein content of *C. d. cumanensis* and assess the ability of immunorecognition by a commercial antivenom.

## 2. Results

### 2.1. Isolation of Fractions of C. d. cumanensis 

**Figure 2:**
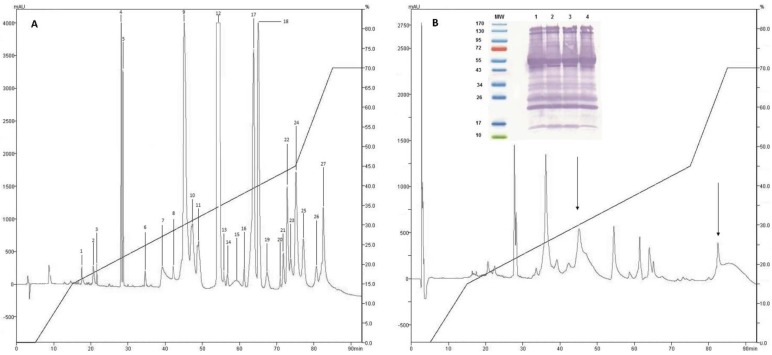
Chromatographic elution profiles by RP-HPLC at 215 nm on a C-18 column. (**A**) Venom pool of *C. d. cumanensis*. (**B**) Complete *C. d. cumanensis* after being treated with Antivipmyn Tri^®^. Internal graphic immunoblot of *C. d. cumanensis* of Colombia. MW: molecular weight marker. 1. Venom pool of *C. d. cumanensis*. 2. Venom of *C. d. cumanensis* from the Caribbean region. 3. Venom of *C. d. cumanensis* from Tolima and Cundinamarca. 4. Venom of *C. d. cumanensis* from Meta.

**Figure 3:**
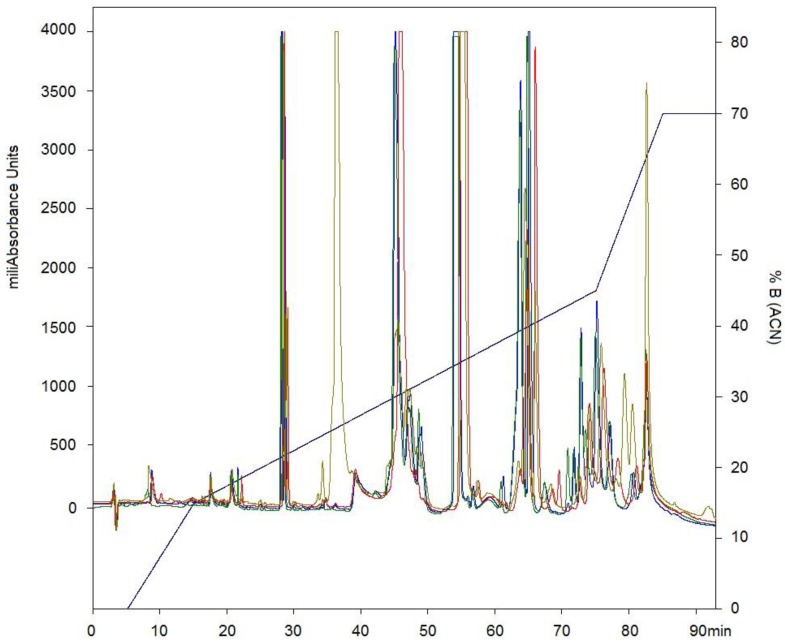
Chromatographic elution profiles by RP-HPLC at 215 nm on a C-18 column. Line blue pool of venom of *C. d. cumanensis*. Line green venom of *C. d. cumanensis* from Meta. Line gray venom of *C. d. cumanensis* from the Caribbean region. Line red venom of *C. d. cumanensis* from Tolima and Cundinamarca.

After separation by RP-HPLC of the complete venom of *C. d. cumanensis*, 27 peaks were observed (Figure 2A). A similar number of peak fractions in the venom pool was observed after separation under the same conditions independently (Figure 3). The chromatographic profiles of *C. d. cumanensis* venom from three different Colombian locations were similar, except the peak RT at 38–40 min of the Caribbean region venom (Figure 3). The identities of some peaks were verified by MS/MS. The peak RT at 38–40 min was shown to be similar to the 9th peak of pool venom and contained crotamine and a PLA_2_. Minor differences in the height of the peaks were observed, suggesting that the three venoms are similar in composition but not in concentration.

### 2.2. Western Blotting and Immunodepletion

Recognition of the venom proteins of *C. d. cumanensis* by the antivenom Antivipmyn Tri^®^ (Instituto Bioclón S.A. de C.V, México City, México) demonstrated that the venoms of the three zones studied (Meta, Cundinamarca-Tolima, and Caribbean coast) did not show significant differences compared to the pool (Figure 2B).

Analysis of the immunodepletion of the complete venom of *C. d. cumanensis* using Antivipmyn Tri^®^ showed reduction in all fractions, which was more significant in fractions containing the complex crotoxin and disintegrins (fractions 1–6). Fractions 20–27 were drastically reduced, which indicates that recognition of proteins of high molecular mass was more effective compared to the crotamine (low molecular mass) present in fractions 8 and 9 (see Figure 2).

### 2.3. Identification of Proteins

**Figure 4:**
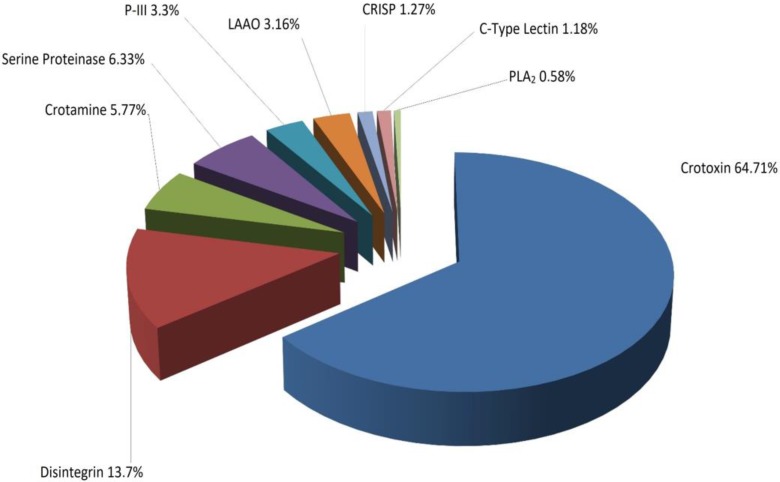
Distribution of whole venom proteins of *C. d. cumanensis* separated by RP-HPLC and identified by nano LC-MS/MS.

Twenty-seven fractions were analyzed by LC/MS digested with trypsin in solution and were subjected to tandem mass analysis, which identified nine groups of proteins whose molecular mass was determined by mass spectrometry–electrospray (ESI) (Table 1). Analysis of the different fractions obtained showed that 64.71% of the venom corresponded to the crotoxin complex (crotoxin A and B), while 13.7% corresponded to disintegrins, as shown in Figure 4. 

## 3. Discussion

Snake venoms are a complex mixture of proteins that induce various signs and symptoms and, in some cases, can lead to the death of the patient by neurotoxicity, as in the case of *C. d. cumanensis* (rattlesnake) [4,7,8]. The use of antivenoms for more than 100 years has allowed for a reduction in the number of deaths associated with these accidents; however, the rapid initiation of symptoms and the delay in starting treatment make it difficult for antivenoms to work properly [15,16]. On the other hand, variability in the venoms is associated with a decrease in neutralizing power between different geographical zones where the same species can be found. In fact, in the *Daboia russelii* species, there is variation in the venom, and thus treatment with an antivenom produced with Indian species is not effective for envenoming by this snake on the island of Sri Lanka [17].

Numerous works have shown variations in the protein composition of the venoms of different snake species and intraspecifically, with respect to ontogeny, season, diet, sex, and the geographical area where the snake is sourced [18,19,20,21,22,23]. The studies have included analyzing variability with respect to the production, toxicity, cross-reactivity, and phylogenetic relationships of venoms, as well as the implications of these differences for clinical management of accidents in consideration of diagnosis, therapy, and production of antivenoms [24,25,26,27,28]. The results obtained with antivenom in immunodepletion and Western blot show immunorecognition of the proteins of *C. d. cumanensis* venom, although this recognition does not imply good neutralization of the effects of the venom in vivo; thus, it is necessary to carry out neutralization tests in vivo.

The rattlesnake species of South America are similar in that their venoms are neurotoxic [1]. Boldrini et al. found that three Brazilian rattlesnake subspecies showed changes in the content of the crotoxin complex, which was 67.4% for *C. d collilineatus* and 72.5% for *C. d. cascavella* [29]. These data are in agreement with those reported in this paper, which show that the content is 64.71% for the same complex. In addition, it is observed that the contents of other proteins, such as LAAO, disintegrins, and serine proteinases, are higher in *C. d. cumanensis* than in *C. d collilineatus* and *C. d. cascavella*, which vary by 2.6% and 0.1% for LAAO, 0.2% and 0.5% for disintegrins, and between 1.9% and 0.5% for serine proteinases [29]. From a clinical point of view, this increase in serine proteinase and disintegrins could be crucial to understanding the coagulant effect of these species due to the action they exert on fibrinogen and platelets, respectively [30].

The content of the crotoxin complex in *C. d. cumanensis* of Venezuela is 2.6% [31], which differs greatly with the results obtained in this work. This was probably because the lethality of *C. d. cumanensis* from Venezuela differs from that observed in this work, since the LD_50_ of the venom from Colombian snakes is lower (160 μg/kg Venezuelan, 50 μg/kg Colombian) [32]; however, it is almost twice as much [32] as that of *C. d. terrificus* of Brazil (65 μg/kg). Previously, Cespedes et al. reported an LD_50_ for the venom of *C. d. cumanensis* of 47 µg/kg for females and 41 µg/kg for juveniles. These results are consistent with those described in this study; however, the males were shown to be less lethal [33].

These differences could be consistent with the content of the crotoxin complex in the different subspecies, or by variations in the venoms that come from different geographical zones. Nonetheless, the same subspecies [1] can have variations according to geographical distribution, age, and some other characteristics that would explain these differences [18,19,20,21,22,23]. Calvete et al. suggested that *C. d. cumanensis* represents the evolutionary transition between northern hemorrhagic rattlesnakes and southern neurotoxic rattlesnakes; however, the results on concentration of crotoxin and lethal dose in this study do not support this evolutionary hypothesis [31].

For development of the work, as well as experimental phases with animals in the treatment of systemic hemorrhage, Antivipmyn Tri^®^ was used, since it demonstrated safety and efficacy for bites by *B. asper* in Colombia [34,35]. Using Western blot, this antivenom demonstrated a high degree of recognition of the proteins in the poisons and a decrease in the proteins of different chromatographic fractions obtained by RP-HPLC, showing that both in vivo and in vitro tests can be used as evaluation methods. Antivenoms can be used to determine the cross-seroreactivity of antivenoms, although preclinical efficacy must ultimately be demonstrated by neutralization tests of toxic effects in animal models.

## 4. Conclusions

In conclusion, knowing the proteomes of the venoms of the different species on the continent would facilitate the search for “universal” antivenoms, since one could deduce or presume equivalence between venoms that could effectively protect the populations of the continent and allow for unification of production standards; however, the lack of knowledge of the venoms of the species in each country makes it difficult to fulfill this goal. For this reason, recent efforts have focused on determining the protein content of American snake species [21,29,36,37,38,39,40,41,42]. This lack of knowledge of venoms is an adverse factor that urges us to carry out tests with different regional antivenoms before suggesting the use of a common antivenom for a single genus, as seen in the different studies carried out in recent years [16,34,43,44,45,46,47].

## 5. Materials and Methods 

### 5.1. Venoms, Chemical Products, and Reagents

From specimens kept in the serpentarium of the University of Antioquia (Medellin, Colombia) and included in the COLBIOFAR-149 collection registered with the Alexander Von Humboldt Research Institute of Biological Resources, venom of *C. d. cumanensis* was obtained by manual milking of 25 specimens from different areas of Colombia (departments of Meta, Tolima, Cundinamarca, and Magdalena). Once extracted, the venom was centrifuged (3000 rpm, 15 min), and the resulting supernatants were lyophilized and stored at −20 °C until use. Antivenin was used, and Antivipmyn Tri^®^, donated by the Bioclón Institute, was produced by hyperimmunization of horses with *Bothrops* spp., *Lachesis* spp., and *Crotalus* spp. (South American) venoms.

### 5.2. Isolation and Characterization of Venom Proteins

The proteins of the complete venom of *C. d. cumanensis* and venom from 3 Colombian locations—Meta state (east region), Caribbean region, and Tolima and Cundinamaca states (central region)—were separated by RP-HPLC on a C-18 RESTEK column (250 mm × 4.6 mm, 5 μm particle size; RESTEK, Bellefonte, PA, USA) with protein detection at 215 nm. The resulting fractions were collected and dried in a speed-vac (Eppendorf, Hamburg, Ham, Germany), and the relative abundance of proteins was estimated from the sum of the aerial chromatographic fractions obtained from the total venom [48,49].

### 5.3. Electrophoresis and Determination of Molecular Mass

Venom (*C. d. cumanensis*) and proteins from each of the obtained fractions were separated under nonreducing conditions by 12% SDS-PAGE [50] and stained with Coomassie Brilliant Blue G-250. The molecular mass of each peak was estimated according to markers (range 97.4 to 14.4 kDa; BioRad, Philadelphia, PA, USA) and confirmed by direct infusion in an ESI electrospray mass spectrometer (IonTrap 6310 series spectrometer, Agilent Technologies, Santa Clara, CA, USA). The molecular mass was deduced by deconvolution using ChemStation V software (version 5.1, Agilent Technologies, Santa Clara, CA, USA).

### 5.4. Identification of Proteins by HPLC-nESI-MS/MS

The isolated fractions of *C. d. cumanensis* were alkylated, reduced, and digested with 0.1 ng trypsin (Agilent Technologies, Santa Clara, CA, USA) at 30 °C overnight. The products of the digestion were then injected into an LC/MS/MS system (1200 series, Agilent Technologies, Santa Clara, CA, USA) on a nano C-18 column (Agilent Zorbax 300SB-C18; 150 × 0.075 mm, 3.5 μm) at a flow of 0.2 μL/min and coupled to an MSD IonTrap mass spectrometer (6310 series, Agilent Technologies, Santa Clara, CA, USA). MS/MS mass spectra were obtained under the following conditions: positive mode, dynamic range from 200 to 1200 Da, electrospray at 2 kV, 230 °C drying temperature, and actuator trap at 200 μs. The ChemStation program G2070-91126 (Agilent Technologies, Santa Clara, CA, USA) was used for deconvolution of the MS/MS spectra in the loaded state. 

### 5.5. Immunodepletion of Venom Proteins by a Mexican Polyvalent Antivenom

Two milligrams of whole venom was dissolved in 70 μL of 20 mM phosphate buffer, pH 7.0, mixed with 4 mg of Antivipmyn TRI^®^ and incubated with gentle stirring overnight at 37 °C. Thereafter, 6 mg of rabbit anti-horse IgG antiserum (Sigma, San Luis, MO, U.S.A) in 350 μL of 20 mM phosphate buffer, pH 7.0, was added, and the mixture was incubated for another 2 h at 37 °C. Immunocomplexes were precipitated by centrifugation at 13,000 rpm for 30 min in an Eppendorf centrifuge, and the supernatant was submitted to reverse-phase separation as described for the isolation of venom proteins. HPLC fractions were characterized as described above. The control sample was subjected to the same procedure, except that antivenom IgGs were not included in the reaction mixture [21,29,36,37,38,39,40,41,42].

### 5.6. Search Database 

The identified peptides obtained from digestion were subjected to a BLAST search [51] to compare with other snake venom protein families. This was performed in BLASTP, and search parameters included nonredundant protein sequence (nr) and snake organism.

### 5.7. BLAST Search of the Identified Peptides

Deconvoluted profile spectra were used to search Mascot [52] and Spectrum Mill (Agilent Technologies, Santa Clara, CA, USA) online in the National Center for Biotechnology Information nr database for protein identification. The parameters of the search included digestion with trypsin, and carbamidomethylation modified (C) as fixed modification with carbamyl (C), carbamyl (N-terminal), carboximethylation (C), oxidation (HW), and oxidation (M) as variable modifications. The minimum score for the intensity of each fraction was 50%, while monoisotopic mass and a mass tolerance of 2.5 Da were used to determine identity. Confirmation of the different peptides was carried out through the selection “Require bold red.”

## Figures and Tables

**Table 1:** Identities of the fractions isolated by RP-HPLC from *C. d. cumanensis*, as shown in Figure 2. Molecular mass determined by nESI, monoisotopic mass of the peptides and their charge, and sequences determined by MS/MS in tandem.

HPLC Fraction	Molecular Mass	Peptide Ion		MS/MS Sequence	Score		Protein Family
		Monoisotopic Mass	Z		Spectrum Mill	Mascot	
1	7591.4	2.036.852	3	LRPGAQCADGLCCDQCR	15.75	42	Disintegrin accession number SP|A2CJE7
2	7431.3	2.036.852	3	LRPGAQCADGLCCDQCR	20		Disintegrin accession number SP|P21858.1
		2.242.927	3	IARGDDMDDYCNGISAGCPR	13.31		
3	7874.2	2.036.852	3	LRPGAQCADGLCCDQCR	19.9		Disintegrin accession number SP|A2CJE7
		1.902.705	3	GDDMDDYCNGISAGCPR	12.7		
4	7545.6	2.050.867	2	LRPGAQCAEGLCCDQCR	18.89	27	Disintegrin accession number SP|P21858.1
5	7762.9	2.050.867	3	LRPGAQCAEGLCCDQCR	17.25		Disintegrin accession number SP|P21858.1
6	7703.7	2.036.852	3	LRPGAQCADGLCCDQCR	19.45	42	Disintegrin accession number SP|A2CJE7
		1.954.729	3	GDWNDDTCTGQSADCPR	14.33		
		1.230.730	2	EAGEECDCGTPGNPCCDAATCK		53	
7	10,730.4	1.549.533	2	CCFEHDCCYAK	15.7	35	Crotoxin acid chain accession number SP|P08878
		1.661.748	3	LTGCDPTTDVYTYR	22.08		
8	4809.9	864.349	2	MDCPWR	6.61	30	Crotamine accession number SP|P24331
		1220.105	2	ICIPPSSDFGK	7.13	41	
9	4810.2	1477.736	3	EKICIPPSSDFGK	11.5	61	Crotamine accession number SP|P24331
		1220.105	2	ICIPPSSDFGK	6.43	69	
	13,856.2	1137.419	2	NCLEESEPC	12.29		PLA_2_-01 accession number GB|BAA08383.1
10	10,319.1	1549.533	3	CCFEHDCCYAK	15.7	20	Crotoxin acid chain accession number SP|P08878
		808.413	2	AAAICFR	12.22	35	
		1294.425	2	GGHGRPQDASDR		19	
		1539.185	2	FSPENCQGESQPC		54	
11	10,400.6	1549.533	3	CCFEHDCCYAK	12.56	14	Crotoxin acid chain accession number SP|P08878
		808.413	2	AAAICFR	9.54	25	
		1539.185	2	FSPENCQGESQPC		48	
12	7502.4	2036.852	2	LRPGAQCADGLCCDQCR	19.9		Disintegrin accession number SP|A2CJE7
	10040.7	1549.533	3	CCFEHDCCYAK	14.43	59	Crotoxin acid chain accession number SP|P08878
13	10,043.2	1549.533	3	CCFEHDCCYAK	11.31		Crotoxin acid chain accession number SP|P08878
		2278.725	2	SSYGCYCGAGGQGWPQDASDR		57	
		808.413	2	AAAICFR		31	
14	10,538.0	1661.748	2	LTGCDPTTDVYTYR	19.39	44	Crotoxin acid chain accession number SP|P08878
		808.413	2	AAAICFR		33	
		2278.725	2	SSYGCYCGAGGQGWPQDASDR		61	
15	14,538.0	1298.551	2	CCFVHDCCYGK	15.89	40	Crotoxin basic chain accession number SP|P62022
		1297.825	2	YGYMFYPDSR	6.35	45	
16	13,970.6	2287.021	3	KNAIPFYAFYGCYCGWGGR	21.42	44	Crotoxin basic chain accession number SP|P62022
		1687.815	3	CNTKWDIYPYSLK	21.08		
		1297.825	2	YGYMFYPDSR	12.53	54	
		1977.905	3	SLSTYKYGYMFYPDSR	20.01	49	
		966.377	2	CRGPSETC	7.5		
17	14,464.4	1687.815	3	CNTKWDIYPYSLK	15.76	43	Crotoxin basic chain accession number SP|P62022
		1505.543	3	CCFVHDCCYGK	14.39	47	
	10,925.4	1549.533	2	CCFEHDCCYAK	8.69		Crotoxin acid chain accession number SP|P08878
	25,435.6	2202.999	3	YSYFYVCQYCPAGNIIGK	12.13		Cysteine-rich secretory protein accession number GB|ACE73575.1
18	13,993.8	1505.543	3	CCFVHDCCYGK	11.31	26	Crotoxin basic chain accession number SP|P62022
		1297.825	2	YGYMFYPDSR		54	
	29,842.0	2890.425	3	LDSPVSDSEHIAPLSLPSSPPSVGSVCR	18.46		Serine proteinase accession number SP|Q5W959
	27,298.5	2291.145	3	NSAHIEPLSLPSSPPSVGSVCR	13.59		Serine proteinase accession number SP|Q71QJ2
19	29,420.1	2142.042	2	LLDDAVCQPPYPELPATSR	13.76	33	Kallikrein-like accession number SP|Q8QHK2
		1068.825	2	EKFFCPNK		30	
		1390.704	3	TLPDVPYCANIK	6.51	34	
20	14,123.2	1505.543	2	CCFVHDCCYGK	15.57	26	Crotoxin basic chain accession number SP|P62022
21	14,735.6	1505.543	3	CCFVHDCCYGK	11.41		Crotoxin basic chain accession number SP|P62022
22	17,789.3	1278.578	2	LWNDQVCESK	10	62	C-type lectin accession number SP|P84987
		1916.891	3	YGESLEIAEYISDYHK	9	56	
23	14,319.1	1505.543	3	CCFVHDCCYGK	13.43		Crotoxin basic chain accession number SP|P62022
	27,292.3	1136.57	2	SVQFDKEQR	5.61		Serine proteinase gyroxin-like accession number GB|ABY65930
24	56,513.5	1236.658	2	SAAQLYVESLR	16.19	72	LAAO accession number SP|P56742
		1165.698	2	IKFEPPLPPK	8.82	32	
		970.522	2	VQVHFNAR	8.87	51	
		2065.063	3	DCADIVINDLSLIHELPK	10.22		
25	54,609.0	1236.658	2	SAAQLYVESLR	16.33	76	LAAO accession number SP|P56742
		2065.063	3	DCADIVINDLSLIHELPK	8.94		
		1222.567	2	DWYANLGPMR	6.97		
26	51,346.6	1851.008	3	KKHDNAQLLTAIDLDR	15.17		P-III metalloproteinase accession number GB|ACV83931
		1160.693	2	FVELVLVVDK	15.94	71	
		2052.055	3	ITVKPEAGYTLNAFGEWR	12.2		
27	49,476.6	1160.693	2	FVELVLVVDK	16.51	68	P-III metalloproteinase accession number GB|ACV83931
		1570.753	3	ENGNKIPCAPEDVK	9.12		
		1052.425	2	GNYYGYCR	9.05		
